# The Gut-Wrenching Effects of Cryptosporidiosis and Giardiasis in Children

**DOI:** 10.3390/microorganisms11092323

**Published:** 2023-09-15

**Authors:** Mayuri Prabakaran, Lyssa J. Weible, Joshua D. Champlain, Ryan Ye Jiang, Katalina Biondi, Ana A. Weil, Wesley C. Van Voorhis, Kayode K. Ojo

**Affiliations:** 1 Center for Emerging and Reemerging Infectious Diseases (CERID), Division of Allergy and Infectious Diseases, Department of Medicine, University of Washington, Seattle, WA 98109, USA; prabmay@uw.edu (M.P.); lyssaw8@uw.edu (L.J.W.); josh419@uw.edu (J.D.C.); rjiang2@uw.edu (R.Y.J.); anaweil@uw.edu (A.A.W.); wesley@uw.edu (W.C.V.V.); 2 Human Center for Artificial Intelligence, Department of Computer Science, University of Central Florida, Orlando, FL 32816, USA; kbiondi@ucf.edu

**Keywords:** children’s gut health, gut microbiota, cryptosporidiosis and giardiasis, undernutrition, immunocompromised

## Abstract

*Cryptosporidium* species and *Giardia duodenalis* are infectious intestinal protozoan pathogens that cause alarming rates of morbidity and mortality worldwide. Children are more likely to have clinical symptoms due to their less developed immune systems and factors such as undernutrition, especially in low- and middle-income countries. The severity of the symptoms and clinical manifestations in children may vary from asymptomatic to life-threatening depending on the *Cryptosporidium* species/*G. duodenalis* strains and the resulting complex stepwise interactions between the parasite, the host nutritional and immunologic status, and the gut microbiome profile. Structural damages inflicted by both parasites to epithelial cells in the large and small intestines could severely impair children’s gut health, including the ability to absorb nutrients, resulting in stunted growth, diminished neurocognitive development, and other long-term effects. Clinically approved cryptosporidiosis and giardiasis drugs have broad antimicrobial effects that have incomprehensible impacts on growing children’s gut health.

## 1. Introduction

Approximately 1.6 million deaths per year are attributed to diarrheal diseases globally. The most recent Global Burden of Disease (GBD) study placed diarrhea as the third leading cause of the increase in disability-adjusted life-years (DALYs) and death [1]. Clinical disease can cause injury to the intestinal lining and alter the intestinal microbiota due to diarrhea-inducing bacteria, viruses, and protozoan parasites [2]. Among the most common bacterial causes of diarrhea in children are *Shigella* species and *Escherichia coli*, which can either be enteropathogenic, enterotoxigenic, enteroinvasive, or Shiga-toxin-producing strains [3,4]. The treatment of bacteria-induced diarrhea includes the use of antibiotics, such as macrolides (azithromycin), glycopeptides (vancomycin), cephalosporins (ceftriaxone), and fluoroquinolones (ciprofloxacin), which are active against different diarrheal pathogens [3,4,5]. A challenge to antibiotic treatment is the potential presence of antimicrobial resistance leading to treatment failure, and the alteration of the gut microbiome, which may have subsequent metabolic and inflammatory effects [6]. Rotavirus-induced diarrhea is the leading cause of severe childhood gastroenteritis and a major contributor to the global burden of diarrheal diseases in infants and children under the age of five years [2,7]. Antiviral therapy has not been developed, but the global prevalence of rotavirus gastroenteritis has been massively reduced by universal rotavirus vaccination [8].

Infectious protozoan parasites are a major cause of severe diarrhea in infants and young children worldwide [9,10]. A disproportionate burden of childhood diarrhea morbidity and mortality caused by infectious protozoan parasites is shouldered by low- and middle-income countries (LMICs), especially among children with undernutrition. A systematic review of 46 studies conducted in 19 African countries from 2005 to 2020 reported that the prevalence of these diseases in children 5–17 years old increased from 19.4% in the first 5-year timeframe to 25.2% in the final year [11]. Among these studies, *Giardia* and *Cryptosporidium* were the second and third most common parasites detected [11]. The Global Enteric Multicenter Study (GEMS) of enteropathogens and other multicenter studies also demonstrated that *Cryptosporidium* and *Giardia* were frequently detected in the stools of children across seven sites in sub-Saharan Africa and South Asia [10,12,13,14]. These studies consistently demonstrated that *Cryptosporidium* was an important cause of pediatric diarrhea, but this was less so for *Giardia*. Co-infection with both *Cryptosporidium* and *Giardia*, or in combination with other pathogens, has been reported in studies from several LMICs [15,16,17]. Infectious protozoan parasites are ubiquitous in the environment and can be found in food, water, and soil. Overall dietary quality, environmental impact, reduced access to urgent medical services, and the quality of healthcare facilities are other reasons for the higher burden of diarrheal diseases in LMICs versus high-income countries (HICs). Unlike bacterial gastroenteritis that can be treated with a variety of antibiotics (and alternative options in cases of resistance), or rotavirus-induced diarrhea that can be prevented with vaccination, cryptosporidiosis and giardiasis have limited treatment options and no clinically approved vaccines. *Cryptosporidium* and *Giardia* infections are challenging to prevent, and the clinical symptoms require specific medications to treat, they are therefore of high public health importance. A large burden of diarrheal diseases of children under the age of 5 years in the world’s poorest countries is due to infections with *Cryptosporidium* and/or *Giardia*. This article will be focused on these infections and their impact on overall gut health in children.

## 2. The Causative Parasites

### 2.1. Cryptosporidium Species

**Figure 1:**
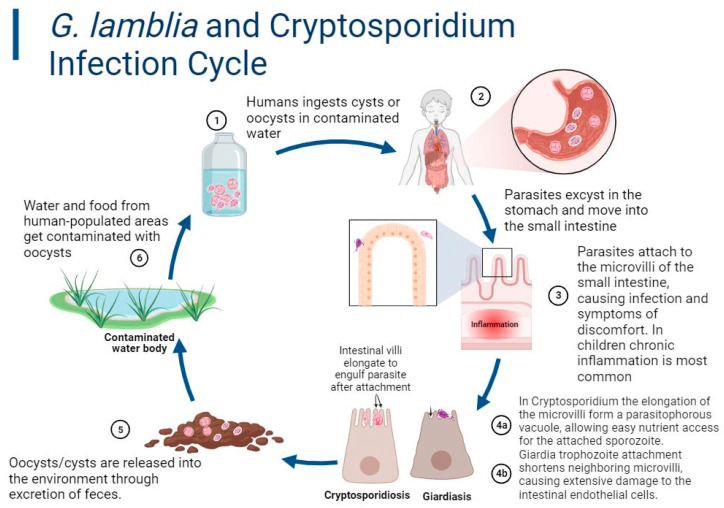
This is a simplified figure showing similarities in the life-cycle stages that initiate human infection and outbreaks of *Cryptosporidium* and *G. duodenalis*. The figure also shows the differences in their impacts on epithelial cells as a result of infection. Adapted from “*Cryptosporidium* Infection Cycle” by Biorender.com (28 August 2023).

Cryptosporidiosis is caused by protozoans in the genus *Cryptosporidium*, which are unicellular, obligate, intracellular parasites. *Cryptosporidium* species reside in intracellular, extracytosolic, parasitophorous vacuoles underneath the apical plasma membranes of the infected intestinal epithelial cells of mammalian hosts [18]. There are at least 44 validated species of *Cryptosporidium* [19]. The morphological differentiation of *Cryptosporidium* based on oocysts or cell analysis is not definitive for species identification [20,21]. Therefore, many species of *Cryptosporidium* were earlier thought to be *C. parvum*, which was first described in the small intestines of mice and subsequently in other mammals. Many of the *Cryptosporidium* species have been known to infect non-human organisms. *C. galli* and *C. avian* genotype VI have been found in avian species. *C. bovis*, *C. ryanae*, and *C. andersoni* have been known to infect cattle. *C. felis* is mostly known to infect cats, and *C. canis* has been found in multiple dog species. *C. melagridis* was described in turkeys. Advances in molecular epidemiologic tools and improved genotypic characterization have helped in identifying more than 20 *Cryptosporidium* species and genotypes in humans. Human infection is nonetheless predominantly caused by *C. parvum* and *C. hominis,* although *C. felis, C. meleagridis, C. canis,* and *C. muris* have also been associated with human zoonotic diseases [19,21,22,23,24,25,26,27]. Protozoan parasites of the genus *Cryptosporidium* have six developmental stages in their overall life cycle [27,28,29]. The developmental stage that initiates human infection and outbreaks is the environmentally resistant and dormant but infectious oocyst stage. Once oocysts are ingested, they excyst, forming active stages (sporozoites, trophozoites, merozoites, etc.) that invade and replicate in the mammalian host’s small intestine (Figure 1) [27,29,30].

### 2.2. Giardia duodenalis

Evolutionarily, *Giardia* belongs to an early branching group, the excavates, thought to have split from the rest of the eukaryotes before plants and animals diverged. The extreme divergence from other eukaryotes is evident in the parasite’s unique morphology, with its two nuclei and four pairs of flagella that allow its motility and attachment to the intestinal epithelium.

This genus of flagellated protozoans infects humans and several other animals. There are currently eight known species of *Giardia*, which have subtle morphological differences: *G. agilis, G. ardeae, G. psittaci, G. muris, G. microti, G. peramelis, G. cricetidarum,* and *G. duodenalis* [31]. *G*. *duodenalis* is also interchangeably called *G. intestinalis* and *G. lamblia* [32,33]. Recently, *G. duodenalis* has been recognized as the preferred scientific name and *G*. *lamblia* the previous name [33]. Morphological differences between the species are commonly identified by features such as the shape and position of the trophozoite bodies, the size of the ventrolateral disk in comparison to the cell length, as well as ultrastructural features visible via electron microscopy [34]. Only one species in this genus, *G. duodenalis,* causes clinical giardiasis in humans and most mammals, although there is a complex of various assemblages that differ in host specificity. The assemblages are subcategories of *G. duodenalis* characterized by subtle genomic differences but wide mammalian host diversity [35]. The assemblages are named from A to H, and some are further subdivided by genotype. *G*. *duodenalis* strains from assemblages A and B are more common in humans and may also be zoonotic. Assemblage A has three phylogenic clusters, of which AI is the dominant zoonotic genotype. AII primarily infects humans but is sometimes isolated in animals, while AIII is commonly associated with wild ruminants [36]. Although assemblage B is heterogeneous, humans are still predominantly affected [37]. Almost all cases of diarrhea involving *G. duodenalis* assemblages A and B are in young children in LMICs. Most *G*. *duodenalis* assemblages infect animals. Domestic animals, such as dogs, cats, and livestock, are the hosts with the widest range of assemblages A–F. There have been sporadic reports of non-A/non-B assemblages or mixed assemblages detected in humans [38,39,40]. Rats and mice are the primary hosts for assemblage G, and grey seals and gulls are the primary hosts for assemblage H [40]. *Cryptosporidium* and *Giardia* are similar in that they have a non-replicating, transmissible form that is stable in the environment and a replicative form(s) that mainly inhabits the small intestine. Unlike *Cryptosporidium* species, all *G. duodenalis* genotypes have only two morphologically distinct life-cycle stages: a motile, flagellated trophozoite that colonizes the proximal small intestine of the mammalian host, and a dormant but infectious cyst stage that can survive for several months in the environment (Figure 1).

## 3. Transmission and Prevalence

There is an association between the high prevalence of symptomatic cryptosporidiosis/giardiasis and a lack of clean or safe drinking water, sanitation infrastructure, healthcare, and/or access to public health information. Undernutrition, which impairs cellular immunity, and immunodeficiencies increase the risk of infection and developing symptoms [41,42]. In sub-Saharan Africa and Asia, 2.9 and 4.7 million cases of cryptosporidiosis, respectively, are estimated to occur annually in children less than 24 months of age. Globally, it is estimated that cryptosporidiosis could be linked to 50 million diarrheal episodes annually, with over 200,000 *Cryptosporidium*-attributable deaths in < 2-year-old malnourished children from Africa and Asia due to moderate-to-severe diarrhea [43,44,45,46,47]. Meanwhile, giardiasis accrues approximately 200 million global symptomatic cases annually, albeit many cases of *Giardia*-associated acute diarrhea may be linked to co-infection with other microorganisms [44,45,48]. Infections are established after the ingestion of dormant, environmentally resistant infective cysts (*G. duodenalis*) or oocysts (*C. parvum* and *C. hominis*), passed in the feces of mammalian hosts [44,49,50]. The most common method of infection involves the consumption of contaminated food or water (Figure 1). The presumed most common method of *Cryptosporidium* infection for young, malnourished children is an infected household contact. The infectious dose of cysts/oocysts that is needed to infect 50% of humans (ID_50_) varies by parasite strain among healthy adults. For many isolates, <10 cysts/oocysts can result in infection [50,51,52,53], although some have shown ID_50′_s of >80 oocysts [53]. *Cryptosporidium* oocysts and *G. duodenalis* cysts are not only shed in vast numbers during diarrhea but are also shed via asymptomatic colonization [54,55,56].

Human *Cryptosporidium* infection was formerly thought to be a zoonosis, but, over the last few decades, studies have demonstrated that most infections are caused by *C. hominis*, for which humans are the natural host, or by strains of *C. parvum*, which are primarily spread from person to person [18]. A recent prospective study demonstrated person-to-person spread from both asymptomatic and symptomatic infected persons [54]. The hardy, highly infectious oocysts can survive for prolonged periods in the environment and are not killed by chlorine, facilitating transmission via chlorinated water. One study demonstrated that the provision of clean drinking water did not reduce the incidence of cryptosporidiosis [57]. In the U.S., *Cryptosporidium* is the most common cause of illness transmitted by recreational water. The earliest record of *Cryptosporidium* as a waterborne pathogen in the United States was in Braun Station, Texas, where over 2000 individuals were diagnosed with cryptosporidiosis after the contamination of their community water supply [58,59]. The parasites have also been linked to waterborne diarrhea, with the largest U.S. outbreak involving 403,000 cases when the Milwaukee water supply was contaminated with *Cryptosporidium* oocysts [60].

Globally, nearly 200 million people have symptomatic *Giardia* infection annually, with total diagnosed incidence rates of approximately 2–3% in HICs and 20–30% in LMICs [32,45,48,61]. Although LMICs face many challenges in combating the spread of these pathogens, there is also evidence to suggest that *Cryptosporidium* and *Giardia* are a growing health concern in HICs as well. *Cryptosporidium* has been demonstrated to have high zoonotic potential, which contributes to the disease occurrence in HICs. The fact that *Cryptosporidium* can be transmitted from infected bovine offspring to humans illustrates its zoonotic potential [62,63]. While the main form of transmission is from person to person, studies have also shown that the transmission of this pathogen is possible from humans to both lambs and mice [62,63]. Various strains of the parasite have been found in other livestock species, such as sheep, goats, horses, and pigs [44]. *Cryptosporidium* may disproportionately affect people of lower socioeconomic status or those in rural areas with exposure to livestock. Agricultural workers in HICs may have a higher potential for acquiring preventable infectious diseases, similar to people living in LMICs [64].

The primary way that *Giardia* initiates infection is typically through the ingestion of a *Giardia* cyst (Figure 1). The cyst is a non-motile *Giardia* oocyte that is resistant to harsh environmental conditions and can survive in water for 1–4 months until ingested by a mammalian host [55,65]. *G. duodenalis*’s zoonotic potential could also be problematic in HICs. Common household pets such as dogs and cats are known to be effective carriers of the parasite and raise the question of the safety of caring for untested pets in households with immunocompromised individuals and young children [66]. The ubiquitous nature of the infective *G. duodenalis* cysts around dog parks could complicate the control of the transmission from pet to pet. There is, however, conflicting evidence about whether exposure to pets poses a threat to humans. In several studies from New Zealand, exposure to infected dogs was not associated with an increased risk of giardiasis, while, in England, studies have suggested that household pets could be associated with an increased risk of disease [66]. Furthermore, an analysis of the literature data based on recent advances in molecular typing tools suggests that zoonotic giardiasis is not as common as previously acknowledged [67]. Nonetheless, cryptosporidiosis and giardiasis are of both human public health and veterinary importance.

## 4. Pathogenesis and Clinical Presentation

The pathogeneses of *Cryptosporidium* and *Giardia* in children are complex. The molecular mechanisms underlying virulence (or pathogenesis) are not fully understood and involve an interplay of host immune responses, parasite virulence factors, and the health status of the host gut microbial community.

### 4.1. Cryptosporidiosis

*Cryptosporidium* species are obligate, intracellular parasites that can damage host intestinal epithelial cells and disrupt the normal balance of fluids and electrolytes. Infectious *Cryptosporidium* oocysts excyst in the intestinal lumen after ingestion. The sporozoite attaches to the intestinal lining using membrane-associated glycoproteins, including gp40/15, which bind to the target cells’ receptors on the ileal brush border, which then enables entry into the host cells, particularly in the terminal ileum [68,69,70,71,72]. Following attachment, the parasite is engulfed by the host cell, causing the elongation of nearby microvilli. This process establishes the parasitophorous vacuole, located within the host cell’s plasma membrane, and a remodeled host-cell cytoskeleton. Subsequent structural changes in the cell also include an invagination at the vacuole interface, which becomes an essential pathway for nutrient access in the host-cell cytoplasm for the parasite [69,73]. Because the parasite has limited abilities for biosynthesis, this nutrient pathway is important for its survival. The parasite undergoes two rounds of asexual replication, which damages the intestinal epithelium, before entering the sexual cycle to produce oocysts that are passed in feces. This process causes the hyperplasia of intestinal crypt cells and the inflammation of the lamina propria, particularly in immunodeficient patients [74]. This could result in watery diarrhea and dehydration. Similarly, studies on human ileocecal cell lines reported that infection with *C*. *parvum* could induce apoptosis as a host response. The disruption of the intestinal barrier indicated by the increased permeability and absorption of lactose to mannitol has been present in some symptomatic cases of cryptosporidial infection in children and HIV-positive adults [75]. However, some people exposed to *Cryptosporidium* are not infected, others are infected and asymptomatic, and others develop diarrhea or enteritis. As mentioned earlier, clinical progress is influenced by a variety of factors, including mannose-binding lectin (MBL), which may play an important protective role in young children and immunodeficient individuals. Polymorphisms in the MBL2 gene can lead to the low production of MBL, making children more prone to diarrheal diseases, including cryptosporidiosis [76,77,78,79]. Younger children infected with *Cryptosporidium* species sometimes develop a persistent inflammatory response of the mucosal surfaces characterized by peripherally circulating proinflammatory cytokines, such as TNF-ɑ and ILF-8 [80]. These proinflammatory cytokines also attract leukocytes to the intestinal tract, which contribute to the onset of diarrhea [80]. For instance, children living with inflammatory bowel diseases, such as Crohn’s disease, typically have increased TNF-ɑ concentrations that have inhibitory impacts on growth plate chondrocytes. This suggests that immune-deficient children with this disease who are also infected with *Cryptosporidium* could have elevated levels of TNF-ɑ, resulting in stunted growth [81]. Some *Cryptosporidium* species are more virulent based on the resulting severity of the tissue damage and inflammation in the infection [27].

### 4.2. Giardiasis

**Figure 2:**
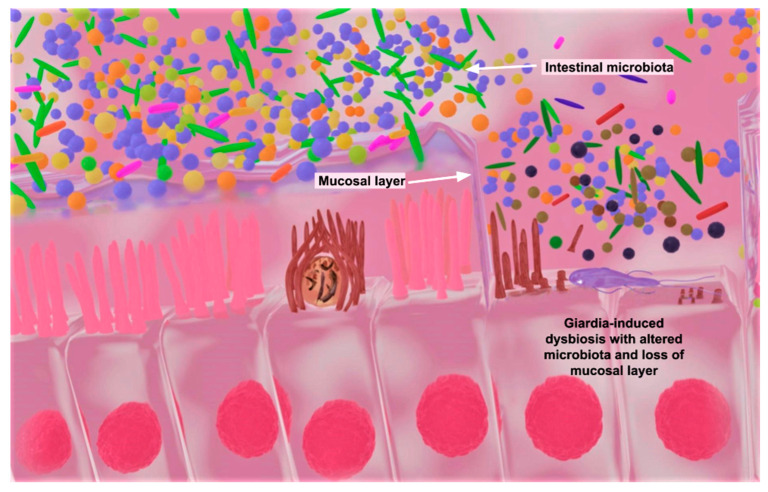
Blender software version 3.5.1-generated image showing a host infected with *Cryptosporidium* (left) and *Giardia* (right). *Cryptosporidium* and/or *G. duodenalis* infection could alter the gut microbiota ecological balance (colored particles) through dysbiosis in the mucosal layers of epithelial cells. *Giardia duodenalis* cysteine proteases, beta-N-acetylglucosaminidase, and beta-N-acetylgalactosaminidase can efficiently break down mucins, which are the molecular framework of the epithelial mucosal layers on the intestine. Histological evidence of the marked depletion of intracellular mucin and gastrointestinal mucosal injury have been reported in HIV-positive individuals with cryptosporidiosis [74].

The molecular mechanisms involved in the pathogenesis of *Giardia* parasites are complex and involve several gene products, including cysteine proteases, variant-specific proteins (VSPs), and adhesion molecules. A simplified version of the molecular mechanisms is presented below. Following ingestion, the *Giardia* cyst makes its way to the stomach and travels to the small intestine. The acidic conditions of the stomach and enzymes from the pancreas are detected by the trophozoites and act as a signal for their emergence from the cyst. Upon excystation in the small intestine, the trophozoite deploys a series of enzymes that help it digest the host’s intestinal proteins. *G. duodenalis* cysteine proteases, beta-N-acetylglucosaminidase, and beta-N-acetylgalactosaminidase play different roles in impairing the natural innate barrier function. They ultimately degrade the intestinal mucus layer, thereby paving the way for *G. duodenalis* access to the epithelial cells and the subsequent attachment to the inner epithelial lining of the intestine [82,83]. *Giardia* replicates in vertebrate hosts after colonization via attachment to the surface of the small intestine with the help of its adhesive ventral disk. Because *G. duodenalis* is non-invasive, it damages the host’s small-intestine epithelial cell lining by inducing an alteration in the expression of tight-junction proteins that maintain the integrity of the intestinal barrier. Cleavage of tight-junction proteins causes increased intestinal permeability, increases in fluid secretion, and decreases in ion absorption, causing a disordered transportation of water and electrolytes within the small intestine, resulting in diarrhea [84,85]. Damages to the mucosal layer and the impairment of the tight-junction integrity alter the epithelial cell defense against intestinal microorganisms, resulting in a variety of immune responses followed by an increase in proinflammatory cytokines [86]. Inflammation of the gut is common due to the redox disruptions caused by varying abundances of Gamma and Beta proteobacteria. These bacteria are considered facultative anaerobes that are sensitive to redox potentials. Increased oxygen tension in the gut anaerobic environment is generally associated with inflammatory induction in the host. Modifications of bacterial biofilms can also increase the production of proinflammatory cytokines, which can occur in giardiasis and, to some extent, in cryptosporidiosis [87,88]. The ensuing intrusions and immune responses may cause mucosal oxidative stress, potentially altering the host microbiota, which could be more devastating in children [89]. The *Giardia* induction of Th17-cell immune responses is associated with increased IL-17 levels in both children and murine models. IL-17 is critical in the immune response to this parasitism, as it is a key cytokine that links T-cell activation to neutrophil activation and mobilization [90,91]. IL-17A also acts as a mediator in antimicrobial activities and transports immunoglobulin A (IgA) across the epithelium to the intestinal lumen. IgA transport creates a chemical barrier that is critical in the clearance of *Giardia*, as IgA targets non-variable antigens produced by *Giardia*. The resulting impairment of the host’s ability to effectively absorb necessary nutrients within the small and large intestines contributes to undernutrition and a shift in the gut flora ecological balance [92,93,94]. Other long-term effects from the pathologic intrusion include intestinal epithelial cell apoptosis, the shortening of the microvilli of intestinal cells, and decreased surface areas of intestinal epithelial cells from the loss of microvilli and villi [92] (Figure 2).

The resulting immune responses, barrier responses, enzyme deficiencies, and shortening of the microvilli can cause many acute and chronic symptoms, which can be especially dangerous for children’s gut health and overall development. A lack of IgA can prolong a *Giardia* infection, and stimulating *Giardia*-specific IgA is a primary area of research for giardiasis vaccine development.

## 5. Symptoms and Clinical Manifestation

During the initial six months of life, most human infants receive much of their passive immunity from breast milk, which can be useful in reducing *Cryptosporidium* and *Giardia* pathologic effects. In addition, the composition and diversity of the gut microbial community can play a crucial role in regulating parasites’ colonization and modulating the host immune responses. As part of the physiological process associated with growth, the infant’s gut microbiota undergoes several changes that can be influenced by genetic, nutritional, and environmental factors and are vastly different from one child to the other. The proper establishment of a healthy microbiota community at an early age can therefore determine the degree of susceptibility and/or resistance to intestinal illnesses through efficient cell signaling directed by microbial flora metabolic byproducts [95]. Abnormal alterations in intestinal bacterial metabolic activities can affect cryptosporidiosis and giardiasis diarrhea progression based on the degree of gut dysbiosis, or microbial imbalance, in the host [91,96]. It is nonetheless common for infected children to have asymptomatic cryptosporidiosis and giardiasis. The molecular, microbiota, and immunologic determinants of asymptomatic cryptosporidiosis and giardiasis are not fully understood. However, variability in immunity or virulence and other factors, including the parasite load, may play roles in the severity after infection, ranging from severe clinical manifestations to asymptomatic infection [58,97].

Conversely, infections with parasites such as *Giardia* and, less significantly, *Cryptosporidium* during childhood may pose a risk of intestinal mucosal disruption causing a shift in the gut community microbial ecological balances, leading to impaired immune responses in the gut (Figure 2). Dysbiosis can specifically cause increased inflammation and tissue damage, which may worsen the severity of cryptosporidiosis and giardiasis diarrhea [91,96]. Cryptosporidiosis and giardiasis illness can not only be self-limited but can also be acute or chronic and persistent [92,98,99,100,101]. These infections have similar clinical symptoms, including diarrhea, abdominal pain, vomiting, and fever. The impact on children can be particularly profound, as both parasitic infections can contribute to malnutrition, stunted growth, and poor cognitive function, with severe cases causing potentially fatal dehydration [10,12,94]. Asymptomatic presentations of the two parasitic infections and the overlapping symptomatology with other parasitic diarrheal diseases are significant public health challenges for which there are limited available diagnostic, treatment, or preventative remedies in hyperendemic LMICs. Although they share some similar symptoms, there are significant differences between cryptosporidiosis and giardiasis in terms of their molecular pathogeneses, pathophysiologies, and overall clinical manifestations of infection (Table 1).

Cryptosporidial diarrhea may be life-threatening and unremitting in some patients, such as children with compromised immune functions, malnutrition, severe HIV infection, or other immunodeficiencies. In immunocompetent persons, cryptosporidiosis diarrhea lasts longer than most infectious diarrhea, often persisting for more than 10 days. Clinical manifestations of *Cryptosporidium* infection sometimes do not include diarrhea but can manifest beyond the GI tract, including by causing respiratory tract diseases and with cough, croup, shortness of breath, hoarseness, and wheezing [28].

There are conflicting conclusions on the role of *G. duodenalis* as the etiologic agent of diarrhea [106,107]. In the GEMS cohorts, *Giardia* infection was associated with an altered gut microbiome in children with and without diarrhea [12]. A follow-up study showed no consistent association between *Giardia* infection and diarrheal symptoms [13]. Other studies demonstrate that giardiasis can cause moderate-to-severe diarrhea, flatulence, weight loss, stomach cramps, and bloating, while chronic infections have been linked with chronic fatigue, long-term irritable bowel syndrome, colitis, food allergies, intestinal permeability, and stunted growth in children [106,108,109,110,111]. Acute symptom onset usually occurs within 1–2 weeks after infection. These symptoms usually resolve within four weeks without treatment; however, chronic infections may persist and could cause potentially dangerous weight loss and vitamin deficiency [56]. Less than half of infected persons develop symptoms in human challenge studies, and asymptomatic infection is common [112]. The severity of the symptoms may be determined by many factors, including the *G. duodenalis* strain’s assemblage, subgenotypes, virulence factor expression, and other factors, such as the antigenic variability of the parasite [32,113,114]. For instance, the pathophysiological damage to the epithelial permeability due to the alteration of intestinal tight junctions observed in many cases could be *G. duodenalis* strain-dependent [115]. Some *G. duodenalis* strains have evolved mechanisms to evade host immune responses by altering the expression of their surface antigens, VSPs, which modulate the specific immune responses mounted by each patient, leading to different clinical outcomes. Antigen variability has also been cited as an impediment to *Giardia* vaccine development [116]. This is because trophozoite lysis by specific antibodies can limit parasite invasion, which can differ based on genotype [37,117]. In addition, lactose intolerance can develop after infection due to epithelial surface damage, further impairing nutrition [112].

## 6. Diagnosis

The accurate diagnosis of cryptosporidiosis and giardiasis is important for prevention and treatment. Diagnostic tests differ in sensitivity and specificity depending on the testing mechanisms and the stage of infection. For the majority of the population affected by these parasites, inexpensive tests and rapid results are essential for prompt therapy. Cryptosporidiosis and giardiasis are both often diagnosed through the microscopic identification of the cysts and oocysts found in fecal matter. However, antigen detection and molecular diagnosis are emerging and are likely to be relatively simple to perform and beneficial in places with limited numbers of trained microscopists [118,119].

Microscopic examination is most frequently performed on stool samples from patients experiencing prolonged diarrhea, abdominal cramping, and vomiting. While used, this method is not very sensitive for either parasite. Cysts and oocysts are concentrated using NaCl flotation and observed using a wet mount under a light or phase-contrast microscope. For easier identification, staining can be used to distinguish parasites from fecal debris. *Giardia* cysts and trophozoites are visualized using iodine, methylene blue, or Giemsa staining [120]. *Cryptosporidium* oocysts in stools are visualized with modified Ziehl–Neelsen (ZN) stains, while Giemsa staining in *Cryptosporidium* diagnostics is used primarily in histopathologic biopsies. Immunological assays yield higher sensitivity and specificity results and use fluorescently labeled antibodies with microscopy to detect parasites.

Antibodies with enzyme reporters in ELISA kits are commercially available and allow for the processing of samples within 10–15 min [121]. Rapid (antigen) diagnostic tests (RDTs) are available for *Giardia* and *Cryptosporidium*, generally using lateral flow techniques. RDTs have been associated with a better correlation to the *Cryptosporidium* causality of diarrhea vs. molecular testing, which can be too sensitive, resulting in false positives due to asymptomatic colonization [122]. Protocols for antibody detection in serum, saliva, and fecal matter can be used to determine the presence of IgA, IgG, and IgM for both parasitic infections. Positive results could reflect current or past infection. Children infected with *Giardia* have increased IgA and IgA responses in their serum and saliva samples compared to other infected and uninfected children [120]. In one study, one-year-old children who developed high fecal IgA levels directed to *Cryptosporidium* Cp23 sporozoite protein were less likely to have delayed growth and recurring infections compared to children with low fecal IgA levels to the same antigen [123].

Molecular methods are not used to diagnose patients with *Giardia* in clinics; however, these methods are used to diagnose *Cryptosporidium* using DNA detection. PCR-based molecular testing is under development in diagnostic laboratories to reduce false negatives [121]. PCR methods that are used in *Giardia* are primarily research-based for the study of different assemblages and sub-types in strains [120]. Biofire and other commercial nucleic acid detection methods for the simultaneous detection of stool pathogens, including *Giardia* and *Cryptosporidium*, have recently replaced microscopy, culture, and antigen diagnostics for stools in many HIC clinical diagnostic laboratories [124]. Multiplex nucleic acid tests are now the most sensitive diagnostic methods; however, in some cases, they are oversensitive. In order to determine whether acute diarrhea is associated with *Cryptosporidium*, a cutoff minimal organism concentration or low PCR cycle time will often be applied [47].

With new developments in technology and medical advances, diagnostic tests are under reevaluation for neglected infectious diseases, such as *Giardia* and *Cryptosporidium*.

## 7. Current Therapy for Cryptosporidiosis and Giardiasis

Despite the great public health concern about the high prevalence among children, the treatment of symptomatic and asymptomatic cryptosporidiosis and giardiasis is often constrained by limited therapeutic options. The efficacy of the currently available antiparasitic agents is suboptimal. In addition, there is no standard dose regimen for persons with immunodeficiencies [125]. Nitazoxanide is the only Food and Drug Administration (FDA)-approved drug with some dual therapeutic effect for the treatment of human and animal cryptosporidiosis and giardiasis (Table 2) [126]. It is also the only FDA-approved drug for the treatment of cryptosporidiosis [47,126,127], and in a randomized trial, it was shown to lead to 2 fewer days of diarrhea compared to untreated patients with cryptosporidiosis [128]. Clinical experience with severely immunocompromised patients and malnourished children has demonstrated frequent therapeutic failure [129,130]. Indeed, effective nitazoxanide therapy may be mediated by the stimulation of innate immune responses that lead to interferon production and antiparasite immune responses [131,132]. This may not be effective in malnourished or immunocompromised individuals. Treatment should also include supportive care, including standard fluid and electrolyte replacement. The avoidance of foods that will exacerbate diarrhea, those high in sodium or glucose, will help to manage the illness [5]. Additionally, improving a child’s diet with supplements would help reduce the symptoms. A deficiency in vitamins, especially zinc, negatively impacts the immune system response. Zinc is vital for the normal function of lymphocytes, including T cells, and improves intestinal epithelium regeneration. Zinc supplements have been shown to reduce the length and severity of diarrhea in children [133,134]. Loperamide is a nonprescription antidiarrheal drug sometimes used as supportive therapy for patients with cryptosporidiosis and giardiasis, especially those with excessive diarrhea [127]. Loperamide, however, is not recommended for use in children due to serious adverse events in infants and toddlers (less than 3 years) [135]. The need for antiparasitic treatment stems beyond just humans to animals, as veterinary practices are a critical point in mitigating the spread of zoonotic diseases.

Food and Drug Administration (FDA)-approved treatments for giardiasis include metronidazole and other 5-nitroimidazoles, as well as the benzimidazole; albendazole (Table 2). However, a substantial number of clinical presentations involve metronidazole (and/or other 5-nitroimidazole drugs, such as tinidazole and secnidazole)-resistant *Giardia* [136,137,138,139]. Second-line drugs such as albendazole, nitazoxanide, furazolidone, and paromomycin also have lower efficacy than nitroimidazoles and/or potentially dangerous side effects [7]. Quinacrine is effective at treating some metronidazole-resistant giardiasis but was withdrawn from the U.S. market due to poor tolerability and limited demand [140,141]. A combined treatment regimen of metronidazole and albendazole or quinacrine can be effective for patients with metronidazole-resistant giardiasis [140,142]. In addition to these widely discussed limitations, some first- and second-line giardiasis drugs (metronidazole, secnidazole, nitazoxanide, paromomycin, and tinidazole) have broad-spectrum antimicrobial activity, which may lead to the development of gut dysbiosis [143,144,145,146]. As discussed above, gut dysbiosis has the potential to cause severe long-term complications in children. The urgency of this public health problem necessitates the accelerated development of alternative drugs for clinical use against *Giardia* and *Cryptosporidium* strains, including those with resistance to current treatments.

**Table 2:** Common treatments available for pediatric cryptosporidiosis and giardiasis with recommended doses.

Drug	Recommended Dose for Children	Duration of Treatment	Side Effects	References
Nitazoxanide *	100–200 mg per 12 h	3 days	Hives; difficulty breathing; swelling of throat, tongue, eyes, and face; headache; stomachache; nausea; discolored urine.	[147]
Metronidazole ^#^	15 mg/kg per day	3 times a day for 5 days	Dizziness; headache; vomiting/nausea; diarrhea; stomach cramps; loss of appetite; dry mouth and metallic taste in mouth.	[148]
Tinidazole ^#^	50 mg/kg per day	1 dose	Nausea/vomiting; diarrhea; bitter or metallic taste in mouth; abdominal pain.	[149,150]
Secnidazole ^#^	30 mg/kg	1 or 2 doses	Gastrointestinal tract disorders similar to Tinidazole.	[151]
Albendazole ^#^	10 mg/kg per day	5 days	Abdominal pain; nausea/vomiting; headache.	[152]
Mebendazole ^#^	100 mg	2 times a day for 3 days	Loss of appetite; abdominal pain; diarrhea, flatulence; nausea/vomiting; headache; tinnitus.	[153]

^#^ Drug for treatment of Giardiasis. * Drug for treatment of cryptosporidiosis and giardiasis.

## 8. Conclusions and Future Outlook

Overall, a complete understanding of the mechanistic links between the host immune system, pediatric gut microbiota development, and establishment of symptomatic *Cryptosporidium* and/or *Giardia* infection is still lacking. The dramatic difference between the LIMC and HIC prevalence of protozoan parasite-induced diarrhea demonstrates the need for more strategic interventions. While creating clean water availability and addressing sanitation infrastructure shortcomings would be ideal, this solution will not come as rapidly as needed to combat these diseases. The establishment of surveillance programs using accurate testing kits that are accessible is essential for the better management/treatment and/or prevention of outbreaks in children living in poverty. In addition, other effective countermeasures are needed to address the threat posed by these parasites in the event of intentional release. Providing medical interventions that are safe, affordable, and accessible with improved efficacy will be a critical step in finding solutions that work. Therefore, evaluation of new therapies should include an assessment of their physiological impact on the gut normal flora, and preference should be given to therapies that do not result in an altered microbiota. In conclusion, further discussions on the impact of *Cryptosporidium* and *Giardia* infection and the drugs used for their treatment on children’s native gut microbial and overall health should be encouraged as part of the public health discussion.

## Figures and Tables

**Table 1:** A comparison of *Giardia* vs. *Cryptosporidium* infection in children at a glance.

Some Differences		Similarities	References
*Giardia*	*Cryptosporidium*		
Microbial biofilm disruption, altered species diversity, and abundance in intestinal microbiota.	No significant changes in the bacterial microbiota in diversity or structure.	Diarrhea is a common associated symptom.	[91,102]
Increased cases more often in children aged 3–6 months old.	More common in children aged 6–12 months old.	Infection lasts longer in children than adults with treatment.	[62,91,103]
Children are 17.9-fold more sensitive to infection than adults.	Children with *Cryptosporidium* are 10.6-fold more sensitive to the infection than adults.	Could cause malnutrition due to lack of nutrient absorption.	[102,104]
*Giardia* infection in children may cause inflammation of the joints.	Children that are HIV-positive are more susceptible to *Cryptosporidium*.	No significant difference in prevalence between males and females among children.	[91,104,105]

## Data Availability

Not applicable.

## References

[1] Abbafati C., Abbas K.M., Abbasi M., Abbasifard M., Abbasi-Kangevari M., Abbastabar H., Abd-Allah F., Abdelalim A., Abdollahi M., Abdollahpour I. (2020). Global burden of 369 diseases and injuries in 204 countries and territories, 1990–2019: A systematic analysis for the Global Burden of Disease Study 2019. Lancet.

[2] Lanata C.F., Fischer-Walker C.L., Olascoaga A.C., Torres C.X., Aryee M.J., Black R.E., Child Health Epidemiology Reference Group of the World Health Organization and UNICEF (2013). Global causes of diarrheal disease mortality in children <5 years of age: A systematic review. PLoS ONE.

[3] Okeke I.N., Nataro J.P. (2001). Enteroaggregative Escherichia coli. Lancet Infect. Dis..

[4] Bruzzese E., Giannattasio A., Guarino A. (2018). Antibiotic treatment of acute gastroenteritis in children. F1000Research.

[5] Kotloff K.L. (2022). Bacterial diarrhoea. Curr. Opin. Pediatr..

[6] Lynch S.V., Pedersen O. (2016). The Human Intestinal Microbiome in Health and Disease. N. Engl. J. Med..

[7] Parashar U.D., Nelson E.A., Kang G. (2013). Diagnosis, management, and prevention of rotavirus gastroenteritis in children. BMJ.

[8] Burnett E., Jonesteller C.L., Tate J.E., Yen C., Parashar U.D. (2017). Global Impact of Rotavirus Vaccination on Childhood Hospitalizations and Mortality From Diarrhea. J. Infect. Dis..

[9] Donowitz J.R., Alam M., Kabir M., Ma J.Z., Nazib F., Platts-Mills J.A., Bartelt L.A., Haque R., Petri W.A. (2016). A Prospective Longitudinal Cohort to Investigate the Effects of Early Life Giardiasis on Growth and All Cause Diarrhea. Clin. Infect. Dis. Off. Publ. Infect. Dis. Soc. Am..

[10] Platts-Mills J.A., Babji S., Bodhidatta L., Gratz J., Haque R., Havt A., McCormick B.J., McGrath M., Olortegui M.P., Samie A. (2015). Pathogen-specific burdens of community diarrhoea in developing countries: A multisite birth cohort study (MAL-ED). Lancet Glob. Health.

[11] Hajissa K., Islam M.A., Sanyang A.M., Mohamed Z. (2022). Prevalence of intestinal protozoan parasites among school children in africa: A systematic review and meta-analysis. PLoS Neglected Trop. Dis..

[12] Kotloff K.L., Nataro J.P., Blackwelder W.C., Nasrin D., Farag T.H., Panchalingam S., Wu Y., Sow S.O., Sur D., Breiman R.F. (2013). Burden and aetiology of diarrhoeal disease in infants and young children in developing countries (the Global Enteric Multicenter Study, GEMS): A prospective, case-control study. Lancet.

[13] Marcenac P., Traore A., Kim S., Prentice-Mott G., Berendes D.M., Powell H., Kasumba I.N., Nasrin D., Jones J.C.M., Zaman S.M.A. (2023). Giardia Detection and Codetection With Other Enteric Pathogens in Young Children in the Vaccine Impact on Diarrhea in Africa (VIDA) Case-Control Study: 2015–2018. Clin. Infect. Dis. Off. Publ. Infect. Dis. Soc. Am..

[14] Operario D.J., Platts-Mills J.A., Nadan S., Page N., Seheri M., Mphahlele J., Praharaj I., Kang G., Araujo I.T., Leite J.P.G. (2017). Etiology of Severe Acute Watery Diarrhea in Children in the Global Rotavirus Surveillance Network Using Quantitative Polymerase Chain Reaction. J. Infect. Dis..

[15] Abdel-Messih I.A., Wierzba T.F., Abu-Elyazeed R., Ibrahim A.F., Ahmed S.F., Kamal K., Sanders J., Frenck R. (2005). Diarrhea associated with *Cryptosporidium parvum* among young children of the Nile River Delta in Egypt. J. Trop. Pediatr..

[16] Gatei W., Wamae C.N., Mbae C., Waruru A., Mulinge E., Waithera T., Gatika S.M., Kamwati S.K., Revathi G., Hart C.A. (2006). Cryptosporidiosis: Prevalence, genotype analysis, and symptoms associated with infections in children in Kenya. Am. J. Trop. Med. Hyg..

[17] Garzon M., Pereira-da-Silva L., Seixas J., Papoila A.L., Alves M. (2018). Subclinical Enteric Parasitic Infections and Growth Faltering in Infants in Sao Tome, Africa: A Birth Cohort Study. Int. J. Environ. Res. Public Health.

[18] White A.C., Bennett J.E., Dolin R., Blaser M.J. (2015). Cryptosporidium Species. Mandell, Douglas, and Bennett’s Principles and Practice of Infectious Diseases.

[19] Ryan U.M., Feng Y., Fayer R., Xiao L. (2021). Taxonomy and molecular epidemiology of *Cryptosporidium* and Giardia—A 50 year perspective (1971–2021). Int. J. Parasitol..

[20] Cunha F.S., Peralta R.H.S., Peralta J.M. (2019). New insights into the detection and molecular characterization of *Cryptosporidium* with emphasis in Brazilian studies: A review. Rev. Inst. Med. Trop. Sao Paulo.

[21] Xiao L., Feng Y. (2017). Molecular epidemiologic tools for waterborne pathogens *Cryptosporidium* spp. and Giardia duodenalis. Food Waterborne Parasitol..

[22] Chappell C.L., Okhuysen P.C., Langer-Curry R.C., Akiyoshi D.E., Widmer G., Tzipori S. (2011). *Cryptosporidium* meleagridis: Infectivity in healthy adult volunteers. Am. J. Trop. Med. Hyg..

[23] Beser J., Toresson L., Eitrem R., Troell K., Winiecka-Krusnell J., Lebbad M. (2015). Possible zoonotic transmission of *Cryptosporidium felis* in a household. Infect. Ecol. Epidemiol..

[24] Thomson S., Hamilton C.A., Hope J.C., Katzer F., Mabbott N.A., Morrison L.J., Innes E.A. (2017). Bovine cryptosporidiosis: Impact, host-parasite interaction and control strategies. Vet. Res..

[25] Tyzzer E. (1912). *Cryptosporidium parvum* (sp. nov.), a coccidium found in the small intestine of the common mouse. Arch. Für Protistenkd..

[26] Cama V.A., Bern C., Roberts J., Cabrera L., Sterling C.R., Ortega Y., Gilman R.H., Xiao L. (2008). *Cryptosporidium* species and subtypes and clinical manifestations in children, Peru. Emerg. Infect. Dis..

[27] Bouzid M., Hunter P.R., Chalmers R.M., Tyler K.M. (2013). *Cryptosporidium* pathogenicity and virulence. Clin. Microbiol. Rev..

[28] Current W.L., Garcia L.S. (1991). Cryptosporidiosis. Clin. Microbiol. Rev..

[29] Dhal A.K., Panda C., Yun S.I., Mahapatra R.K. (2022). An update on *Cryptosporidium* biology and therapeutic avenues. J. Parasit. Dis..

[30] Boulter-Bitzer J.I., Lee H., Trevors J.T. (2007). Molecular targets for detection and immunotherapy in *Cryptosporidium parvum*. Biotechnol. Adv..

[31] Ryan U., Zahedi A. (2019). Molecular epidemiology of giardiasis from a veterinary perspective. Adv. Parasitol..

[32] Feng Y., Xiao L. (2011). Zoonotic potential and molecular epidemiology of *Giardia* species and giardiasis. Clin. Microbiol. Rev..

[33] Simner P.J. (2017). Medical Parasitology Taxonomy Update: January 2012 to December 2015. J. Clin. Microbiol..

[34] Monis P.T., Thompson R.C. (2003). *Cryptosporidium* and Giardia-zoonoses: Fact or fiction?. Infect. Genet. Evol..

[35] Michaels S.A., Hulverson M.A., Whitman G.R., Tran L.T., Choi R., Fan E., McNamara C.W., Love M.S., Ojo K.K. (2022). Repurposing the Kinase Inhibitor Mavelertinib for Giardiasis Therapy. Antimicrob. Agents Chemother..

[36] Seabolt M.H., Roellig D.M., Konstantinidis K.T. (2022). Genomic comparisons confirm Giardia duodenalis sub-assemblage AII as a unique species. Front. Cell. Infect. Microbiol..

[37] Adam R.D. (2021). Giardia duodenalis: Biology and Pathogenesis. Clin. Microbiol. Rev..

[38] Broglia A., Weitzel T., Harms G., Caccio S.M., Nockler K. (2013). Molecular typing of Giardia duodenalis isolates from German travellers. Parasitol. Res..

[39] Soliman R.H., Fuentes I., Rubio J.M. (2011). Identification of a novel Assemblage B subgenotype and a zoonotic Assemblage C in human isolates of Giardia intestinalis in Egypt. Parasitol. Int..

[40] Heyworth M.F. (2016). Giardia duodenalis genetic assemblages and hosts. Parasite.

[41] Calder P.C., Jackson A.A. (2000). Undernutrition, infection and immune function. Nutr. Res. Rev..

[42] Astiazaran-Garcia H., Inigo-Figueroa G., Quihui-Cota L., Anduro-Corona I. (2015). Crosstalk between Zinc Status and Giardia Infection: A New Approach. Nutrients.

[43] Khalil I.A., Troeger C., Rao P.C., Blacker B.F., Brown A., Brewer T.G., Colombara D.V., De Hostos E.L., Engmann C., Guerrant R.L. (2018). Morbidity, mortality, and long-term consequences associated with diarrhoea from *Cryptosporidium* infection in children younger than 5 years: A meta-analyses study. Lancet Glob. Health.

[44] Squire S.A., Ryan U. (2017). *Cryptosporidium* and *Giardia* in Africa: Current and future challenges. Parasites Vectors.

[45] Pires S.M., Fischer-Walker C.L., Lanata C.F., Devleesschauwer B., Hall A.J., Kirk M.D., Duarte A.S., Black R.E., Angulo F.J. (2015). Aetiology-Specific Estimates of the Global and Regional Incidence and Mortality of Diarrhoeal Diseases Commonly Transmitted through Food. PLoS ONE.

[46] Sow S.O., Muhsen K., Nasrin D., Blackwelder W.C., Wu Y., Farag T.H., Panchalingam S., Sur D., Zaidi A.K., Faruque A.S. (2016). The Burden of *Cryptosporidium* Diarrheal Disease among Children <24 Months of Age in Moderate/High Mortality Regions of Sub-Saharan Africa and South Asia, Utilizing Data from the Global Enteric Multicenter Study (GEMS). PLoS Neglected Trop. Dis..

[47] Gilbert I.H., Vinayak S., Striepen B., Manjunatha U.H., Khalil I.A., Van Voorhis W.C. (2023). Safe and effective treatments are needed for cryptosporidiosis, a truly neglected tropical disease. BMJ Glob. Health.

[48] Hajare S.T., Chekol Y., Chauhan N.M. (2022). Assessment of prevalence of *Giardia lamblia* infection and its associated factors among government elementary school children from Sidama zone, SNNPR, Ethiopia. PLoS ONE.

[49] Todd E., Motarjemi Y. (2014). Foodborne Diseases: Overview of Biological Hazards and Foodborne Diseases.

[50] Busatti H.G., Santos J.F., Gomes M.A. (2009). The old and new therapeutic approaches to the treatment of giardiasis: Where are we?. Biologics.

[51] DuPont H.L., Chappell C.L., Sterling C.R., Okhuysen P.C., Rose J.B., Jakubowski W. (1995). The infectivity of *Cryptosporidium parvum* in healthy volunteers. N. Engl. J. Med..

[52] Okhuysen P.C., Chappell C.L., Crabb J.H., Sterling C.R., DuPont H.L. (1999). Virulence of three distinct *Cryptosporidium parvum* isolates for healthy adults. J. Infect. Dis..

[53] Chappell C.L., Okhuysen P.C., Langer-Curry R., Widmer G., Akiyoshi D.E., Tanriverdi S., Tzipori S. (2006). *Cryptosporidium hominis*: Experimental challenge of healthy adults. Am. J. Trop. Med. Hyg..

[54] Korpe P., Ni Z.M., Kabir M., Alam M., Ferdous T., Ara R., Munday R.M., Haque R., Duggal P. (2023). Prospective Cohort Study of *Cryptosporidium* Infection and Shedding in Infants and Their Households. Clin. Infect. Dis..

[55] Adam R.D. (2001). Biology of *Giardia lamblia*. Clin. Microbiol. Rev..

[56] Dunn N., Juergens A.L. (2022). Giardiasis. StatPearls [Internet].

[57] Sarkar R., Ajjampur S.S.R., Prabakaran A.D., Geetha J.C., Sowmyanarayanan T.V., Kane A., Duara J., Muliyil J., Balraj V., Naumova E.N. (2013). Cryptosporidiosis Among Children in an Endemic Semiurban Community in Southern India: Does a Protected Drinking Water Source Decrease Infection?. Clin. Infect. Dis..

[58] D’Antonio R.G., Winn R.E., Taylor J.P., Gustafson T.L., Current W.L., Rhodes M.M., Gary G.W., Zajac R.A. (1985). A waterborne outbreak of cryptosporidiosis in normal hosts. Ann. Intern. Med..

[59] Graczyk T.K., Fayer R., Cranfield M.R. (1997). Zoonotic transmission of *Cryptosporidium parvum*: Implications for water-borne cryptosporidiosis. Parasitol. Today.

[60] Mac Kenzie W.R., Hoxie N.J., Proctor M.E., Gradus M.S., Blair K.A., Peterson D.E., Kazmierczak J.J., Addiss D.G., Fox K.R., Rose J.B. (1994). A massive outbreak in Milwaukee of *Cryptosporidium* infection transmitted through the public water supply. N. Engl. J. Med..

[61] Escobedo A.A., Almirall P., Robertson L.J., Franco R.M., Hanevik K., Morch K., Cimerman S. (2010). Giardiasis: The ever-present threat of a neglected disease. Infect. Disord. Drug Targets.

[62] Tzipori S. (1988). Cryptosporidiosis in perspective. Adv. Parasitol..

[63] Bamaiyi P.H., Redhuan N.E.M. (2016). Prevalence and risk factors for cryptosporidiosis: A global, emerging, neglected zoonosis. Asian Biomed..

[64] Suolaniemi J., Autio T., Heikkinen J., Rasanen K. (2023). Knowledge, Attitudes, and Practices of Finnish Dairy Farmers on Cryptosporidiosis. J. Agromed..

[65] Rumsey P., Waseem M. (2022). *Giardia lamblia* Enteritis. StatPearls [Internet].

[66] Bowman D.D., Lucio-Forster A. (2010). Cryptosporidiosis and giardiasis in dogs and cats: Veterinary and public health importance. Exp. Parasitol..

[67] Cai W., Ryan U., Xiao L., Feng Y. (2021). Zoonotic giardiasis: An update. Parasitol. Res..

[68] Winter G., Gooley A.A., Williams K.L., Slade M.B. (2000). Characterization of a major sporozoite surface glycoprotein of Cryptosporidum parvum. Funct. Integr. Genom..

[69] Cui Z.H., Wang L.Y., Wang Y.X., Li J., Wang R.J., Sun M.F., Zhang L.X. (2020). *Cryptosporidium parvum* gp40/15 Is Associated with the Parasitophorous Vacuole Membrane and Is a Potential Vaccine Target. Microorganisms.

[70] Tzipori S., Ward H. (2002). Cryptosporidiosis: Biology, pathogenesis and disease. Microbes Infect..

[71] Chen X.M., Keithly J.S., Paya C.V., LaRusso N.F. (2002). Cryptosporidiosis. N. Engl. J. Med..

[72] Borad A., Ward H. (2010). Human immune responses in cryptosporidiosis. Future Microbiol..

[73] Leitch G.J., He Q. (2012). Cryptosporidiosis-an overview. J. Biomed. Res..

[74] Lumadue J.A., Manabe Y.C., Moore R.D., Belitsos P.C., Sears C.L., Clark D.P. (1998). A clinicopathologic analysis of AIDS-related cryptosporidiosis. AIDS.

[75] Ojcius D.M., Perfettini J.L., Bonnin A., Laurent F. (1999). Caspase-dependent apoptosis during infection with *Cryptosporidium parvum*. Microbes Infect..

[76] Kelly P., Jack D.L., Naeem A., Mandanda B., Pollok R.C., Klein N.J., Turner M.W., Farthing M.J. (2000). Mannose-binding lectin is a component of innate mucosal defense against *Cryptosporidium parvum* in AIDS. Gastroenterology.

[77] Kirkpatrick B., Huston C., Wagner D., Noel F., Rouzier P., Pape J., Bois G., Larsson C., Alston W., Tenney K. (2006). Serum mannose-binding lectin deficiency is associated with cryptosporidiosis in young Haitian children. Clin. Infect. Dis..

[78] Carmolli M., Duggal P., Haque R., Lindow J., Mondal D., Petri W.A., Mourningstar P., Larsson C.J., Sreenivasan M., Khan S. (2009). Deficient serum mannose-binding lectin levels and MBL2 polymorphisms increase the risk of single and recurrent *Cryptosporidium* infections in young children. J. Infect. Dis..

[79] Mead J.R. (2023). Early immune and host cell responses to *Cryptosporidium* infection. Front. Parasitol..

[80] Kirkpatrick B.D., Noel F., Rouzier P.D., Powell J.L., Pape J.W., Bois G., Alston W.K., Larsson C.J., Tenney K., Ventrone C. (2006). Childhood cryptosporidiosis is associated with a persistent systemic inflammatory response. Clin. Infect. Dis..

[81] Ballinger A. (2002). Fundamental mechanisms of growth failure in inflammatory bowel disease. Horm. Res. Paediatr..

[82] Amat C.B., Motta J.P., Fekete E., Moreau F., Chadee K., Buret A.G. (2017). Cysteine Protease-Dependent Mucous Disruptions and Differential Mucin Gene Expression in Giardia duodenalis Infection. Am. J. Pathol..

[83] Connaris S., Greenwell P. (1997). Glycosidases in mucin-dwelling protozoans. Glycoconj. J..

[84] Otani T., Furuse M. (2020). Tight Junction Structure and Function Revisited. Trends Cell Biol..

[85] Solaymani-Mohammadi S. (2022). Mucosal Defense Against Giardia at the Intestinal Epithelial Cell Interface. Front. Immunol..

[86] Halliez M.C., Motta J.P., Feener T.D., Guerin G., LeGoff L., Francois A., Colasse E., Favennec L., Gargala G., Lapointe T.K. (2016). Giardia duodenalis induces paracellular bacterial translocation and causes postinfectious visceral hypersensitivity. Am. J. Physiol. Liver Physiol..

[87] Barash N.R., Maloney J.G., Singer S.M., Dawson S.C. (2017). Giardia Alters Commensal Microbial Diversity throughout the Murine Gut. Infect. Immun..

[88] Laurent F., Eckmann L., Savidge T.C., Morgan G., Theodos C., Naciri M., Kagnoff M.F. (1997). *Cryptosporidium parvum* infection of human intestinal epithelial cells induces the polarized secretion of C-X-C chemokines. Infect. Immun..

[89] Circu M.L., Aw T.Y. (2012). Intestinal redox biology and oxidative stress. Semin. Cell Dev. Biol..

[90] Zenobia C., Hajishengallis G. (2015). Basic biology and role of interleukin-17 in immunity and inflammation. Periodontol. 2000.

[91] Fekete E., Allain T., Siddiq A., Sosnowski O., Buret A.G. (2020). *Giardia* spp. and the Gut Microbiota: Dangerous Liaisons. Front. Microbiol..

[92] Halliez M.C., Buret A.G. (2013). Extra-intestinal and long term consequences of Giardia duodenalis infections. World J. Gastroenterol. WJG.

[93] Fletcher S.M., Stark D., Harkness J., Ellis J. (2012). Enteric protozoa in the developed world: A public health perspective. Clin. Microbiol. Rev..

[94] Checkley W., White A.C., Jaganath D., Arrowood M.J., Chalmers R.M., Chen X.M., Fayer R., Griffiths J.K., Guerrant R.L., Hedstrom L. (2015). A review of the global burden, novel diagnostics, therapeutics, and vaccine targets for *Cryptosporidium*. Lancet Infect. Dis..

[95] Weber-Stiehl S., Jarke L., Castrillon-Betancur J.C., Gilbert F., Sommer F. (2022). Mitochondrial Function and Microbial Metabolites as Central Regulators of Intestinal Immune Responses and Cancer. Front. Microbiol..

[96] Hakim H., Dallas R., Wolf J., Tang L., Schultz-Cherry S., Darling V., Johnson C., Karlsson E.A., Chang T.C., Jeha S. (2018). Gut Microbiome Composition Predicts Infection Risk During Chemotherapy in Children With Acute Lymphoblastic Leukemia. Clin. Infect. Dis. Off. Publ. Infect. Dis. Soc. Am..

[97] Gupta V.K., Kim M., Bakshi U., Cunningham K.Y., Davis J.M., Lazaridis K.N., Nelson H., Chia N., Sung J. (2020). A predictive index for health status using species-level gut microbiome profiling. Nat. Commun..

[98] Thompson R.C., Olson M.E., Zhu G., Enomoto S., Abrahamsen M.S., Hijjawi N.S. (2005). *Cryptosporidium* and cryptosporidiosis. Adv. Parasitol..

[99] Feasey N.A., Healey P., Gordon M.A. (2011). Review article: The aetiology, investigation and management of diarrhoea in the HIV-positive patient. Aliment. Pharmacol. Ther..

[100] O’Connor R.M., Shaffie R., Kang G., Ward H.D. (2011). Cryptosporidiosis in patients with HIV/AIDS. AIDS.

[101] Shirley D.A., Moonah S.N., Kotloff K.L. (2012). Burden of disease from cryptosporidiosis. Curr. Opin. Infect. Dis..

[102] Toro-Londono M.A., Bedoya-Urrego K., Garcia-Montoya G.M., Galvan-Diaz A.L., Alzate J.F. (2019). Intestinal parasitic infection alters bacterial gut microbiota in children. PeerJ.

[103] Wegayehu T., Adamu H., Petros B. (2013). Prevalence of Giardia duodenalis and *Cryptosporidium* species infections among children and cattle in North Shewa Zone, Ethiopia. MC Infect. Dis..

[104] Han M.Y., Xiao S.M., An W., Sang C.H., Li H.Y., Ma J.F., Yang M. (2020). Co-infection risk assessment of Giardia and *Cryptosporidium* with HIV considering synergistic effects and age sensitivity using disability-adjusted life years. Water Res..

[105] Gebre B., Alemayehu T., Girma M., Ayalew F., Tadesse B.T., Shemelis T. (2019). Cryptosporidiosis And Other Intestinal Parasitic Infections And Concomitant Threats Among HIV-Infected Children In Southern Ethiopia Receiving First-Line Antiretroviral Therapy. HIV/AIDS Res. Palliat. Care.

[106] Muhsen K., Levine M.M. (2012). A systematic review and meta-analysis of the association between *Giardia lamblia* and endemic pediatric diarrhea in developing countries. Clin. Infect. Dis. Off. Publ. Infect. Dis. Soc. Am..

[107] Michaels S.A., Hennessey K.M., Paragas N., Paredez A.R., Ojo K.K. (2021). A Curious Case for Development of Kinase Inhibitors as Antigiardiasis Treatments Using Advanced Drug Techniques. ACS Infect. Dis..

[108] Dann S.M., Le C.H.Y., Hanson E.M., Ross M.C., Eckmann L. (2018). Giardia Infection of the Small Intestine Induces Chronic Colitis in Genetically Susceptible Hosts. J. Immunol..

[109] Litleskare S., Rortveit G., Eide G.E., Hanevik K., Langeland N., Wensaas K.A. (2018). Prevalence of Irritable Bowel Syndrome and Chronic Fatigue 10 Years After Giardia Infection. Clin. Gastroenterol. Hepatol..

[110] Di Prisco M.C., Hagel I., Lynch N.R., Jimenez J.C., Rojas R., Gil M., Mata E. (1998). Association between giardiasis and allergy. Ann. Allergy Asthma Immunol..

[111] Rogawski E.T., Bartelt L.A., Platts-Mills J.A., Seidman J.C., Samie A., Havt A., Babji S., Trigoso D.R., Qureshi S., Shakoor S. (2017). Determinants and Impact of Giardia Infection in the First 2 Years of Life in the MAL-ED Birth Cohort. J. Pediatr. Infect. Dis. Soc..

[112] Nash T.E., Herrington D.A., Losonsky G.A., Levine M.M. (1987). Experimental human infections with *Giardia lamblia*. J. Infect. Dis..

[113] Homan W.L., Mank T.G. (2001). Human giardiasis: Genotype linked differences in clinical symptomatology. Int. J. Parasitol..

[114] Wang Y., Gonzalez-Moreno O., Roellig D.M., Oliver L., Huguet J., Guo Y., Feng Y., Xiao L. (2019). Epidemiological distribution of genotypes of Giardia duodenalis in humans in Spain. Parasites Vectors.

[115] Chin A.C., Teoh D.A., Scott K.G., Meddings J.B., Macnaughton W.K., Buret A.G. (2002). Strain-dependent induction of enterocyte apoptosis by *Giardia lamblia* disrupts epithelial barrier function in a caspase-3-dependent manner. Infect. Immun..

[116] Davids B.J., Liu C.M., Hanson E.M., Le C.H.Y., Ang J., Hanevik K., Fischer M., Radunovic M., Langeland N., Ferella M. (2019). Identification of Conserved Candidate Vaccine Antigens in the Surface Proteome of *Giardia lamblia*. Infect. Immun..

[117] Faubert G. (2000). Immune response to Giardia duodenalis. Clin. Microbiol. Rev..

[118] Chan R., Chen J., York M.K., Setijono N., Kaplan R.L., Graham F., Tanowitz H.B. (2000). Evaluation of a combination rapid immunoassay for detection of *Giardia* and *Cryptosporidium* antigens. J. Clin. Microbiol..

[119] Garcia L.S., Shimizu R.Y., Novak S., Carroll M., Chan F. (2003). Commercial assay for detection of *Giardia lamblia* and *Cryptosporidium parvum* antigens in human fecal specimens by rapid solid-phase qualitative immunochromatography. J. Clin. Microbiol..

[120] Hooshyar H., Rostamkhani P., Arbabi M., Delavari M. (2019). *Giardia lamblia* infection: Review of current diagnostic strategies. Gastroenterol. Hepatol. Bed Bench.

[121] Khurana S., Chaudhary P. (2018). Laboratory diagnosis of cryptosporidiosis. Trop. Parasitol..

[122] Kabir M., Ahmed E., Hossain B., Alam M., Ahmed S., Taniuchi M., Gilchrist C.A., Houpt E.R., Faruque A., Petri W.A. (2018). Giardia/*Cryptosporidium* QUIK CHEK assay is more specific than quantitative polymerase chain reaction for rapid point-of-care diagnosis of cryptosporidiosis in infants in Bangladesh. Clin. Infect. Dis..

[123] Kabir M., Alam M., Nayak U., Arju T., Hossain B., Tarannum R., Khatun A., White J.A., Ma J.N.Z., Haque R. (2021). Nonsterile immunity to cryptosporidiosis in infants is associated with mucosal IgA against the sporozoite and protection from malnutrition. PLoS Pathog..

[124] Chang L.J., Hsiao C.J., Chen B., Liu T.Y., Ding J., Hsu W.T., Su-Ortiz V., Chen S.T., Su K.Y., Wu H.P. (2021). Accuracy and comparison of two rapid multiplex PCR tests for gastroenteritis pathogens: A systematic review and meta-analysis. BMJ Open Gastroenterol..

[125] Farsi T.A., Weerakoon S., Mohsin J., Al Mashayakhi H., Ahmed K., Al Maani A., Aboqusida K., Al Sukaiti N. (2021). Disseminated Cryptosporidiosis in an Infant with Non-HIV Pediatric Immunodeficiency: First Case Report from Oman. Oman Med. J..

[126] Fox L.M., Saravolatz L.D. (2005). Nitazoxanide: A new thiazolide antiparasitic agent. Clin. Infect. Dis. Off. Publ. Infect. Dis. Soc. Am..

[127] Sparks H., Nair G., Castellanos-Gonzalez A., White A.C. (2015). Treatment of *Cryptosporidium*: What We Know, Gaps, and the Way Forward. Curr. Trop. Med. Rep..

[128] Rossignol J.F., Ayoub A., Ayers M.S. (2001). Treatment of diarrhea caused by *Cryptosporidium parvum*: A prospective randomized, double-blind, placebo-controlled study of Nitazoxanide. J. Infect. Dis..

[129] Amadi B., Mwiya M., Musuku J., Watuka A., Sianongo S., Ayoub A., Kelly P. (2002). Effect of nitazoxanide on morbidity and mortality in Zambian children with cryptosporidiosis: A randomised controlled trial. Lancet.

[130] Amadi B., Mwiya M., Sianongo S., Payne L., Watuka A., Katubulushi M., Kelly P. (2009). High dose prolonged treatment with nitazoxanide is not effective for cryptosporidiosis in HIV positive Zambian children: A randomised controlled trial. BMC Infect. Dis..

[131] Di Santo N., Ehrisman J. (2014). A functional perspective of nitazoxanide as a potential anticancer drug. Mutat. Res. Mol. Mech. Mutagen..

[132] Jasenosky L.D., Cadena C., Mire C.E., Borisevich V., Haridas V., Ranjbar S., Nambu A., Bavari S., Soloveva V., Sadukhan S. (2019). The FDA-Approved Oral Drug Nitazoxanide Amplifies Host Antiviral Responses and Inhibits Ebola Virus. iScience.

[133] Shane A.L., Mody R.K., Crump J.A., Tarr P.I., Steiner T.S., Kotloff K., Langley J.M., Wanke C., Warren C.A., Cheng A.C. (2017). 2017 Infectious Diseases Society of America Clinical Practice Guidelines for the Diagnosis and Management of Infectious Diarrhea. Clin. Infect. Dis. Off. Publ. Infect. Dis. Soc. Am..

[134] Bajait C., Thawani V. (2011). Role of zinc in pediatric diarrhea. Indian J. Pharmacol..

[135] Li S.T., Grossman D.C., Cummings P. (2007). Loperamide therapy for acute diarrhea in children: Systematic review and meta-analysis. PLoS Med..

[136] Nabarro L.E., Lever R.A., Armstrong M., Chiodini P.L. (2015). Increased incidence of nitroimidazole-refractory giardiasis at the Hospital for Tropical Diseases, London: 2008–2013. Clin. Microbiol. Infect..

[137] Farthing M.J. (1996). Giardiasis. Gastroenterol. Clin. N. Am..

[138] Tejman-Yarden N., Eckmann L. (2011). New approaches to the treatment of giardiasis. Curr. Opin. Infect. Dis..

[139] Lalle M., Hanevik K. (2018). Treatment-refractory giardiasis: Challenges and solutions. Infect. Drug Resist..

[140] Leitsch D. (2015). Drug Resistance in the Microaerophilic Parasite *Giardia lamblia*. Curr. Trop. Med. Rep..

[141] Gardner T.B., Hill D.R. (2001). Treatment of giardiasis. Clin. Microbiol. Rev..

[142] Bawa S., Kumar S., Drabu S., Kumar R. (2010). Structural modifications of quinoline-based antimalarial agents: Recent developments. J. Pharm. Bioallied Sci..

[143] Lofmark S., Edlund C., Nord C.E. (2010). Metronidazole is still the drug of choice for treatment of anaerobic infections. Clin. Infect. Dis. Off. Publ. Infect. Dis. Soc. Am..

[144] Riches A., Hart C.J.S., Trenholme K.R., Skinner-Adams T.S. (2020). Anti-Giardia Drug Discovery: Current Status and Gut Feelings. J. Med. Chem..

[145] Ibrahim A.A., El-Housseiny G.S., Aboshanab K.M., Yassien M.A., Hassouna N.A. (2019). Paromomycin production from Streptomyces rimosus NRRL 2455: Statistical optimization and new synergistic antibiotic combinations against multidrug resistant pathogens. BMC Microbiol..

[146] Singh N., Narayan S. (2011). Nitazoxanide A Broad Spectrum Antimicrobial. Med. J. Armed Forces India.

[147] Rossignol J.F., Lopez-Chegne N., Julcamoro L.M., Carrion M.E., Bardin M.C. (2012). Nitazoxanide for the empiric treatment of pediatric infectious diarrhea. Trans. R. Soc. Trop. Med. Hyg..

[148] Standing J.F., Ongas M.O., Ogwang C., Kagwanja N., Murunga S., Mwaringa S., Ali R., Mturi N., Timbwa M., Manyasi C. (2018). Dosing of Ceftriaxone and Metronidazole for Children With Severe Acute Malnutrition. Clin. Pharmacol. Ther..

[149] Speelman P. (1985). Single-Dose Tinidazole for the Treatment of Giardiasis. Antimicrob. Agents Chemother..

[150] Escobedo A.A., Alvarez G., Gonzalez M.E., Almirall P., Canete R., Cimerman S., Ruiz A., Perez R. (2008). The treatment of giardiasis in children: Single-dose tinidazole compared with 3 days of nitazoxanide. Ann. Trop. Med. Parasitol..

[151] Gillis J.C., Wiseman L.R. (1996). Secnidazole. A review of its antimicrobial activity, pharmacokinetic properties and therapeutic use in the management of protozoal infections and bacterial vaginosis. Drugs.

[152] Morch K., Hanevik K. (2020). Giardiasis treatment: An update with a focus on refractory disease. Curr. Opin. Infect. Dis..

[153] Perez F., Vallet T., Bravo Z., Callahan K., Ruiz F. (2021). Acceptability of Mebendazole Chewable Tablet in Children Aged 2 to 4 Years in Peru. Pharmaceutics.

